# Nitric oxide dependent signaling via cyclic GMP in dendritic cells regulates migration and T-cell polarization

**DOI:** 10.1038/s41598-018-29287-9

**Published:** 2018-07-20

**Authors:** Stefanie Gnipp, Evanthia Mergia, Michelle Puschkarow, Albrecht Bufe, Doris Koesling, Marcus Peters

**Affiliations:** 1 Department of Experimental Pneumology, Medical Faculty, Ruhr University Bochum, 44780 Bochum, Germany; 2 Institute of Pharmacology and Toxicology, Medical Faculty, Ruhr University Bochum, 44780 Bochum, Germany

## Abstract

Allergic airway inflammation is accompanied by excessive generation of nitric oxide (NO). Beside its detrimental activity due to the generation of reactive nitrogen species, NO was found to modulate immune responses by activating the NO-sensitive Guanylyl Cyclases (NO-GCs) thereby mediating the formation of the second messenger cyclic GMP (cGMP). To investigate the contribution of the key-enzyme NO-GC on the development of Th2 immunity *in vivo*, we sensitized knock-out (KO) mice of the major isoform NO-GC1 to the model allergen ovalbumin (OVA). The loss of NO-GC1 attenuates the Th2 response leading to a reduction of airway inflammation and IgE production. Further, *in vitro*-generated OVA-presenting DCs of the KO induce only a weak Th2 response in the WT recipient mice upon re-exposure to OVA. *In vitro*, these NO-GC1 KO BMDCs develop a Th1-polarizing phenotype and display increased cyclic AMP (cAMP) formation, which is known to induce Th1-bias. According to our hypothesis of a NO-GC1/cGMP-dependent regulation of cAMP-levels we further demonstrate activity of the cGMP-activated cAMP-degrading phosphodiesterase 2 in DCs. Herewith, we show that activity of NO-GC1 in DCs is important for the magnitude and bias of the Th response in allergic airway disease most likely by counteracting intracellular cAMP.

## Introduction

Dendritic cells (DCs) are the most potent antigen-presenting cells (APCs) and play a pivotal role in the induction of allergic airway disease. Their immune modulatory potency is altered in the presence of pathogen-derived compounds and several soluble mediators which induce the maturation of DCs^1,2^. The DCs lose the ability of phagocytosis during their maturation following antigen-presentation and undergo a change in the expression pattern of chemokine receptors, with the latter allowing the directional movement along chemokine gradients from the peripheral tissues to lymphatic organs^3^. Specifically, the expression of the chemokine receptor CCR7 is induced, which sensitizes the DCs to the chemokines CCL19/CCL21, thereby guiding the DCs to the T-cell regions in the lymph node^4,5^. Microbial danger signals such as lipopolysaccharide (LPS) were shown to induce the maturation of APCs and priming T-cell fate in a dose-dependent fashion. We have shown previously that the Th2-priming competence of DCs depends on treatment with low doses of LPS, whereas high doses trigger a Th17 response^6^. However, it is still unclear which molecular phenotype BMDCs must acquire to orchestrate the induction of a Th2 response. There is contradicting literature about the involvement of surface molecules in Th2 polarization^7–9^. One recent report showed that the deficiency in the formation of the second messenger cyclic AMP (cAMP) in the DCs results in a spontaneous Th2 response even without immunization^10^.

Nitric oxide (NO) is produced by activated innate immune cells via the iNOS^11^. Subsequently, the produced NO can further evolve to different reactive nitrogen species that are able to kill microbes^12–14^. Besides this important protective function of NO it is an important signaling molecule acting via activation of the NO sensitive guanylyl cyclase (NO-GC) leading to the formation of the second messenger cGMP^15^. Here it fulfills essential physiological function like the regulation of the tonus of blood vessels. However cGMP has also been shown to regulate important functions of immune cells such as the maturation and endocytosis of human DCs^16,17^. Beside regulating innate immune cells it was also reported to impact the regulation of the adaptive immune responses^18,19^. In T-cells, NO-dependent signaling via cGMP was found to increase the expression of the receptor IL12Rß2, thereby enhancing their Th1 differentiation^19^. Further it was shown that exogenous NO polarizes human pDCs toward a Th2-promoting phenotype partly via a cGMP-dependent pathway *in vitro*^18^. But neither the contribution of the NO-activated cGMP-generating key-enzyme NO-GC, nor the kind of responsible NO-GC-isoform has been described *in vivo* under pathophysiological condition.

The NO-GCs are heterodimeric enzymes and exist in two isoforms (NO-GC1; NO-GC2) with similar functional properties^20–24^. Expression of NO-GC in the lung is comparatively high, with NO-GC1 as the predominant isoform while NO-GC2 represents less than 10%^25–28^. The NO-GCs catalyze the biosynthesis of intracellular cyclic GMP (cGMP) which activates cGMP effector proteins (kinases, phosphodiesterases: PDEs, ion channels), thereby transducing most of the biological NO effects^15,29^. The cGMP response is terminated by PDEs which degrade cGMP.

Some of the PDEs, such as the cGMP- and cAMP-degrading PDE2, contain regulatory (so-called) GAF domains with a high affinity for binding cGMP which increases the cAMP-degrading catalytic activity of PDE2 notably. Hence, low cGMP levels in tissues containing PDE2 can enhance the degradation of cAMP^30^.

Since NO-dependent signaling via cGMP was shown to modulate the immune response *in vitro* the aim of this study was to investigate how and to what extend the key-enzyme of NO-mediated signaling, the NO-GC is involved in the induction of Th2 immunity *in vivo*. Therefore we used knock-out (KO) mice in which the major isoform NO-GC1 is deleted (NO-GC1 KO). Mice deficient of NO-GC1 are viable, fertile and do not show obvious phenotypical abnormalities. In the present study, we used these mice to elucidate the contribution of NO-induced cGMP effects on sensitization, airway inflammation and Th2-response in an Ovalbumin-based model for allergic airway inflammation. Herewith, we report a bifunctional regulatory role of NO-dependent cGMP signaling via the isoform NO-GC1 in DCs as we found it to be required for both chemotaxis and T-cell polarization in Th2 immunity with evidence for a cGMP/cAMP signaling axis.

## Results

### The Th2 inflammatory response is reduced in NO-GC1-deficient mice in a model of allergic airway inflammation

**Figure 1:**
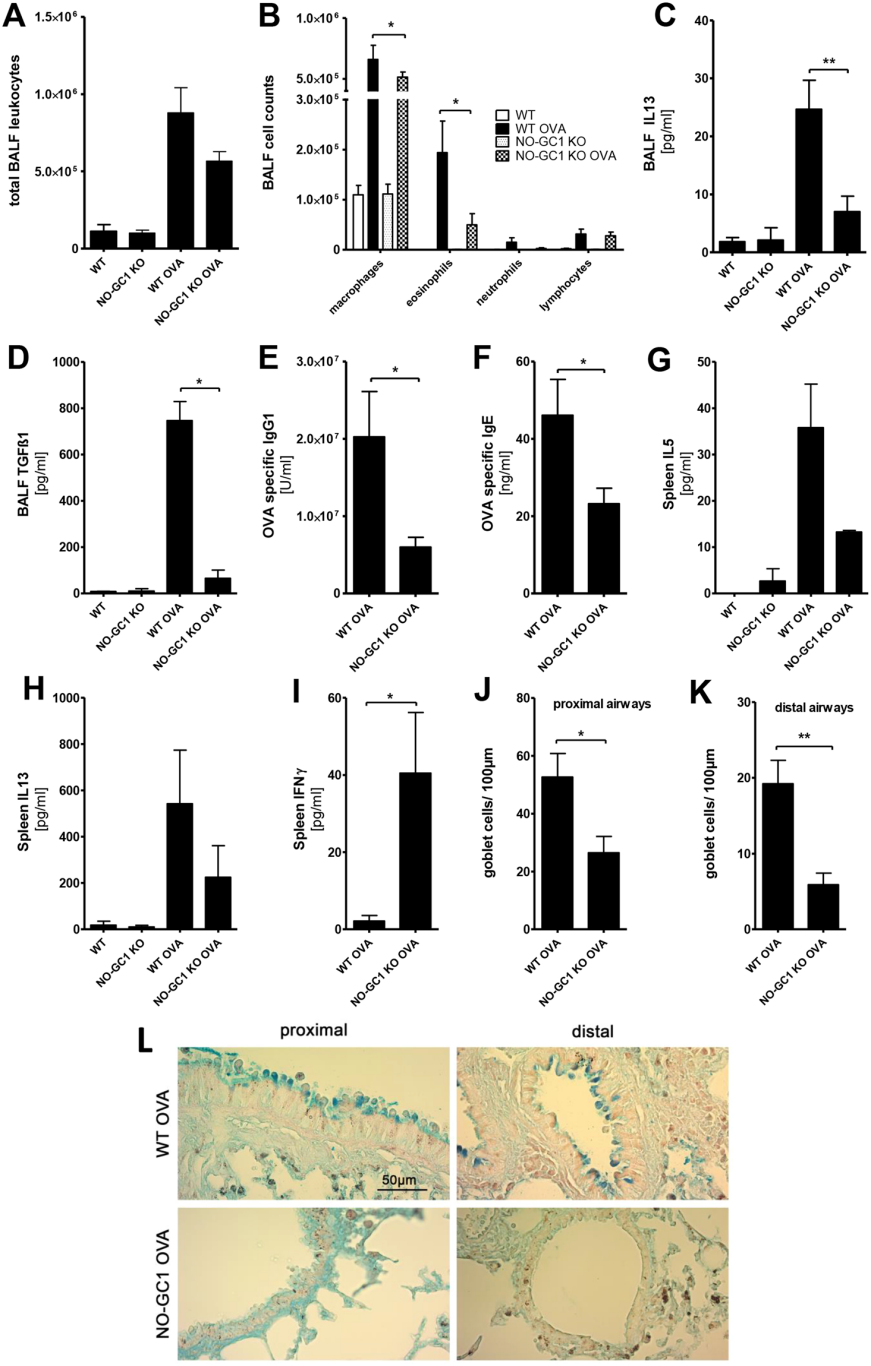
Allergic inflammation in OVA-treated WT and NO-GC1-deficient mice. (**A**) Total cells and (**B**) differential cell count in BAL fluids of OVA-treated and untreated WT and NO-GC1 KO mice. (**C**,**D**) Concentration of IL13 and TGFß1 measured in BAL fluid by means of ELISA. (**E**) OVA-specific IgG1 and (**F**) OVA-specific IgE levels in serum. There was no OVA-specific IgG1 and IgE detectable in the untreated WT and NO-GC1 KO animals. (**G**–**I**) Cytokine response of splenocytes after restimulation with OVA as measured in the cell culture supernatants (ELISA). There was no IFNγ detectable in the splenocytes of untreated WT and NO-GC1 KO animals. (**J**–**L**) The amount of goblet cells related to the lengths of the basement membrane in the proximal (**J** and **L**) and distal (**K** and **L**) airways in periodic acid-Schiff stained tissue (magnification: 40x). Data are mean ± SEM of n = 4 untreated WT and NO-GC1 KO, n = 6 OVA-treated WT and NO-GC1 KO mice for each experiment of two independent experiments in (**C**–**F**). One-way ANOVA with subsequent Dunn’s post test (**A**–**D**,**G**,**H**) or Mann-Whitney test (**E**,**F**,**I**–**K**) was performed. *P < 0.05, **P < 0.01.

We used the established ovalbumin (OVA) model of antigen-driven Th2-dependent acute allergic inflammation to investigate the role of NO-GC1 in the pathogenesis of allergic asthma. Sensitization of wildtype (WT) and NO-GC1 KO with OVA-alum followed by a challenge with OVA aerosol was performed as indicated in the methods section. After the treatment, inflammatory parameters were analyzed in the bronchoalveolar lavage (BAL). As expected, the BAL of OVA-sensitized WT mice displayed a pronounced increase of leukocytes, characterized by the accumulation of eosinophils in the differential cell count, increase of the Th2 cytokine interleukin (IL)13 and the TGFß1 content (Fig. 1A–D). Compared to WT, the BAL of OVA-treated NO-GC1 KOs contained significantly fewer eosinophils (~1/4 of WT treated) and lower levels of IL13 and TGFß1 (~1/2 and 1/10 of WT OVA, respectively). Concentrations of IL4 and IL5 in the BAL were below the detection limit of the ELISA test. We conclude that the inflammatory response induced by the OVA challenge is reduced in the NO-GC1 KOs. Consistent with the alterations in the BAL, OVA-specific IgG1 and OVA-specific IgE were reduced in sera of the NO-GC1 KOs (Fig. 1E,F). Furthermore, we determined the cytokine production (IL5, IL10, IL13, IL17A and IFNγ) upon restimulation of splenocytes with OVA (Fig. 1G–I). Here, the secretion of the Th2 cytokines IL5 and IL13 was found to be reduced by trend (not significant = ns) by about 60% in the NO-GC1 KO, whereas the production of the Th1 cytokine IFNγ was increased. As a common biomarker for the activity of Th17 cells IL17A was not detectable in the supernatants. The production of the anti-inflammatory cytokine IL10 was increased in cells of mice that were sensitized and challenged with OVA; there was no difference however between WT and KO mice (Supplementary Fig. 1E–G). We stained lung sections with periodic acid-Schiff for mucus-producing cells and counted the periodic acid-Schiff positive cells (Fig. 1J–L) to address whether the reduced inflammatory response in the NO-GC1 KOs also results in fewer morphological changes in the bronchial epithelium, such as goblet cell hyperplasia. We found a reduced number of goblet cells (up to threefold) in the lung tissue of OVA-treated NO-GC1 KOs. Yet, airway reactivity to methacholine was similarly increased in both sensitized and OVA-challenged strains (Supplementary Fig. 1A,B). Likewise the inflammation in the lung tissue as evaluated with H&E staining is comparable between the two mouse strains (Supplementary Fig. 1C) suggesting that the overall inflammation is not reduced in the KO mice but shifted to another phenotype. This is further supported by measurement of the Th1 specific transcription factor Tbet in T-helper cells after intraperitioneal sensitization with OVA/Alum. Spleens of wildtype mice contain 2+/−0.9% CD4-positive T cells that do also stain positive for Tbet. After sensitization with OVA/Alum the expression of this transcription factor is not increased significantly (2.6+/−0.9%). Comparable to WT mice NO-GC1 KO mice have 1.7+/−0.7% Tbet-positive T-helper cells. In contrast, due to sensitization with OVA/Alum, the percentage of Tbet expressing CD4 positive T cells rises to 6.4+/−0.6% (p < 0.05 as determined by the Kruskal Wallis Test followed by the Dunn’s post test).

### The T-cell response is reduced and imbalanced in WT mice which received NO-GC1 KO bone marrow-derived DCs for allergic sensitization

We examined the functionality of DCs in an adoptive transfer model to track the precise role of NO-GC1 within the antigen-induced Th2 response. For that purpose, bone marrow (BM) cells were isolated and differentiated *in vitro* to BMDCs^31,32^. This cell type was extensively used to study the sensitization phase during the development of the allergic immune response in the lung^32^. The phenotype of BMDCs resembles inflammatory DCs that are generated from monocytic precursors under inflammatory conditions in the lung. Inflammatory DCs are known to play an important role in allergic sensitization via the airways^33^. The purity and maturation of the BMDCs was confirmed by fluorescence-activated cell sorting (FACS) analysis of major DC marker molecules CD11c, MHCII, the co-stimulatory molecule CD86 and OX40L. Neither the proportion of CD11c+ cells nor the expression of OX40L, CD86 and MHCII was altered in the NO-GC1 KOs compared to WT (Supplementary Fig. 2).

**Figure 2:**
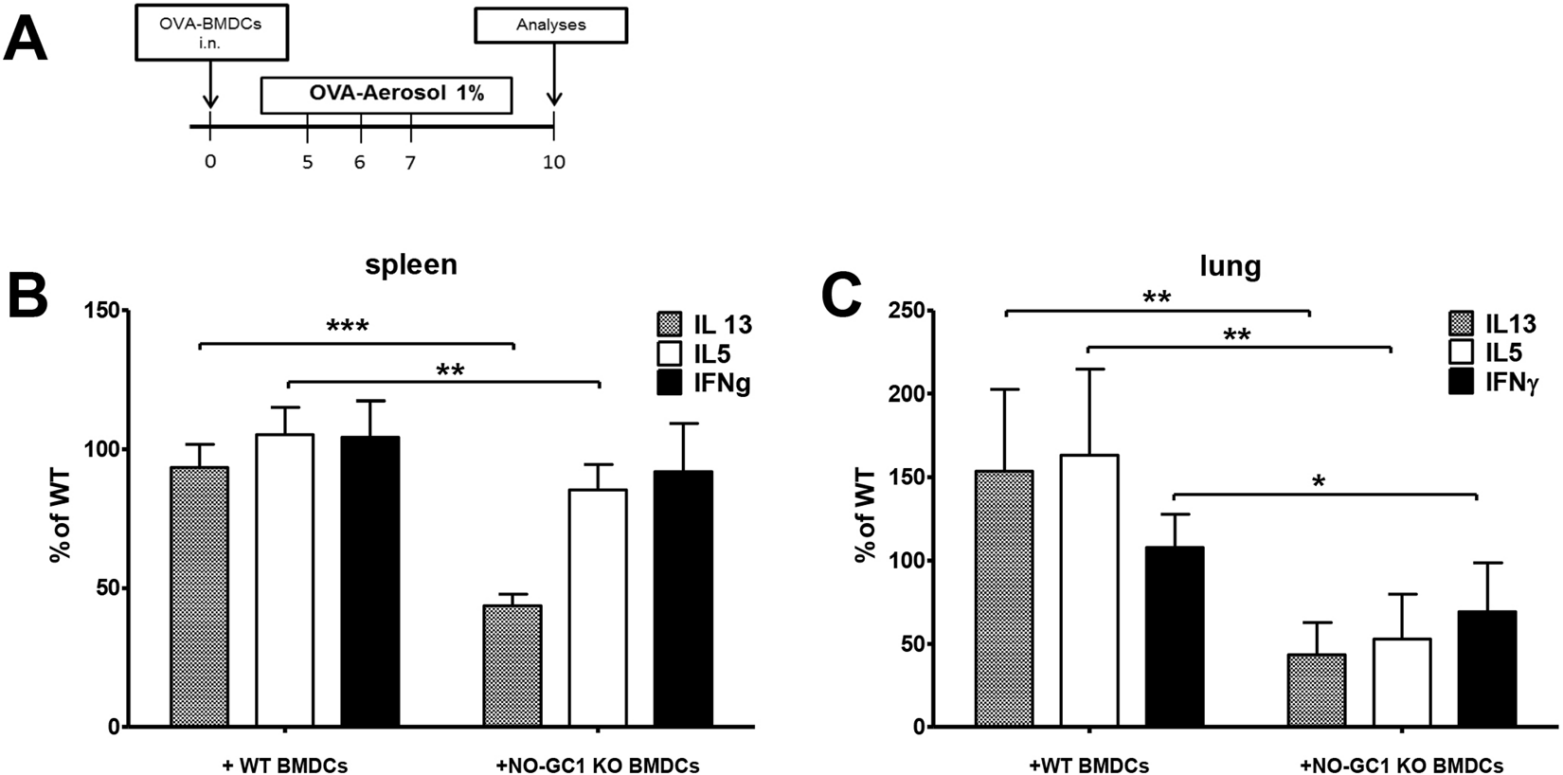
Inflammatory response upon transfer of OVA-pulsed BMDCs from WT and NO-GC1-deficient mice to WT recipient mice. (**A**) Protocol of the adoptive transfer. To analyze their sensitizing capacity, OVA-loaded BMDCs generated from WTs and NO-GC1 KOs were transferred (intranasal) to WT mice. These recipient mice were challenged with OVA-aerosol on three consecutive days and analyzed as indicated. (**B**) Cytokine response of splenocytes and (**C**) lung cells after restimulation with OVA as measured in cell culture supernatants (ELISA). The level of the cytokines analyzed was set as 100% in the WT situation. The cytokines IL5, IL13 and IFNγ were undetectable in control animals. Data are mean ± SEM of n = 4–8 mice/group for each experiment and two independent experiments. To increase the power the data of the two experiments were combined. To account for inter-experimental variation of the total amount of cytokines produced the results are presented as percent increase/decrease of the produced cytokines compared to mice sensitized with WT BMDCs. One-way ANOVA with subsequent Dunn’s post test or Mann-Whitney test were performed. *P < 0.05, **P < 0.01, ***P < 0.001.

The immune competence of allergen-loaded BMDCs from either NO-GC1 KO or WT to induce a Th2 response was analyzed after adoptive transfer to WT mice and subsequent allergen challenge (Fig. 2A). Afterwards the Th2 response of the recipient mice was analyzed *ex vivo* in single cell cultures of spleen and lung. The production of the Th2 cytokines IL5 and IL13 was reduced significantly upon restimulation in the recipients of NO-GC1 KO BMDCs in either splenocytes (Fig. 2B) or lung cells (Fig. 2C). The secretion of the Th1 cytokine IFNγ by splenocytes did not differ (Fig. 2B), however, is significantly reduced when lung cells were studied (Fig. 2C). Strikingly, the relation of Th1/Th2 cytokines is shifted towards Th1 in the recipients of NO-GC1 KO BMDCs, since IFNγ is not reduced to the same extent as IL5 and IL13. The results indicate that attenuation of the Th2 response observed in the NO-GC1 KO is partially due to a reduced Th2-inducing capacity and a Th1 bias of their DCs.

### NO-GC1 deficient BMDCs exhibit an impaired migration due to enhanced expression of endogenous CCL19

**Figure 3:**
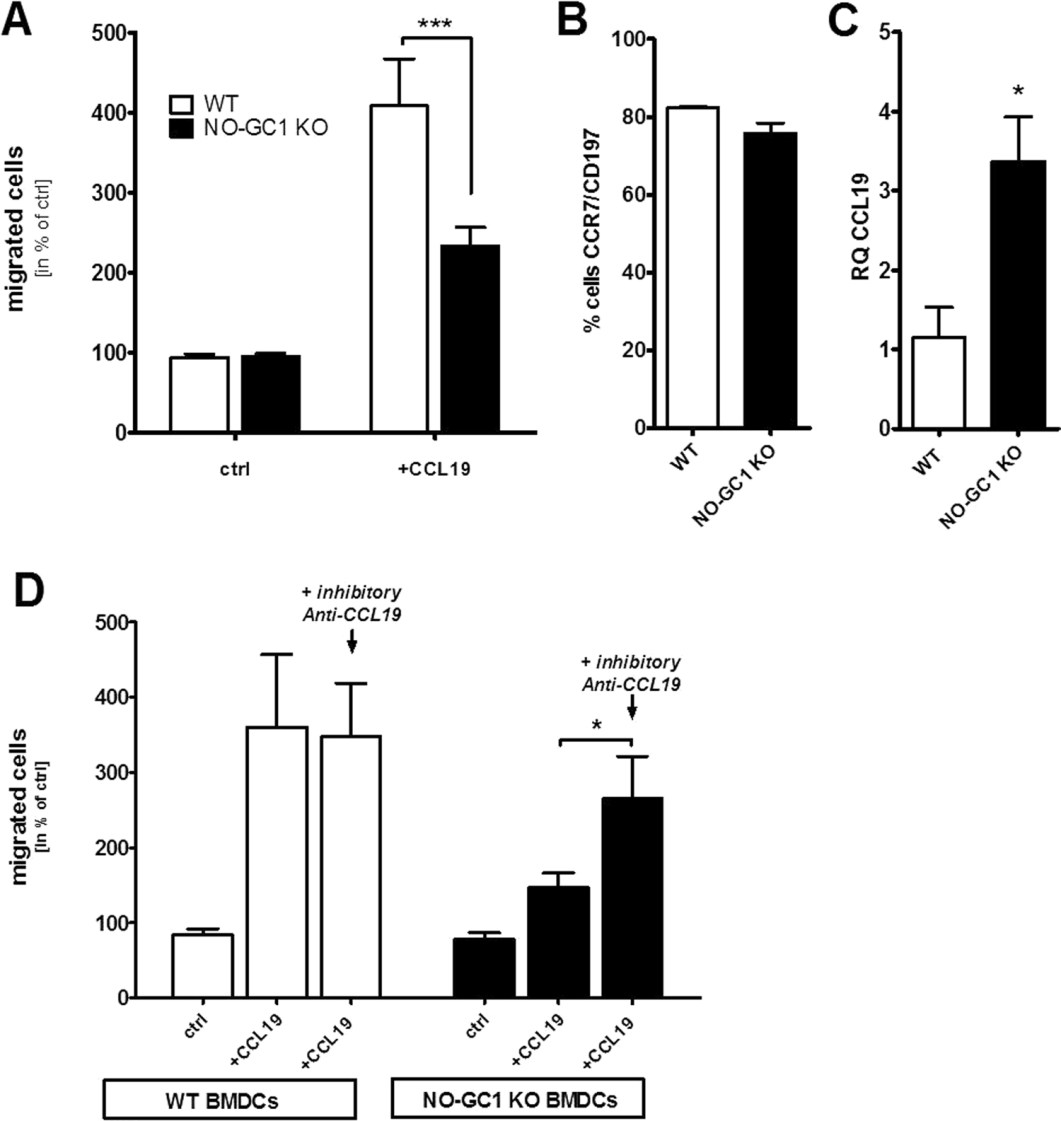
Analysis of migrational properties of BMDCs from WT and NO-GC1-deficient mice. (**A**) Proportion of migrated cells towards CCL19 in the transwell assay. Spontaneous migration (without CCL19) referred to as control (ctrl). (**B**) Relative amount of CCR7/CD197 expressing BMDCs in FACS analysis. (**C**) Relative quantification (RQ) of *CCL19* mRNA expression in BMDCs as measured with Real Time PCR. (**D**) Proportion of migrated BMDCs towards CCL19 with and without 24 h preincubation with a neutralizing anti-CCL19 antibody. Spontaneous migration (without CCL19) referred to as control (ctrl). Data are mean ± SEM of (**A**) n = 9/group from four independent experiments and (**B**–**D**) n = 4–6/group from two independent experiments. Two-way ANOVA with subsequent Bonferroni’s post test or Student’s test were performed. *P < 0.05, **P < 0.01, ***P < 0.001.

We analyzed the migration properties of the NO-GC1 KO BMDCs because the inflammatory response upon the adoptive transfer of the NO-GC1 KO BMDCs was generally reduced. Interestingly, we found an impaired migration of these BMDCs towards the chemokine CCL19 *in vitro*. The proportion of migrated NO-GC1 KO cells in the transwell assay was decreased by about one-third compared to WT (Fig. 3A). Since the chemokine-triggered migration depends on the CCL19/21-receptor CCR7, we investigated its surface expression in FACS analysis. However, the expression of CCR7 was not changed in the NO-GC1 KO (Fig. 3B, representative dot plots of the flow cytometric analysis are shown in Supplementary Fig. 3). By contrast, we found a notably increased expression of CCL19 transcript in the NO-GC1 KO BMDCs compared to the WT cells (Fig. 3C). We analyzed the migration upon inhibition of endogenous CCL19 prior to the transwell assay with a neutralizing anti-CCL19 antibody to test whether the impaired migration of the NO-GC1 KO BMDCs is a result of the enhanced release of CCL19. Indeed, upon neutralization of CCL19, the NO-GC1 KO BMDCs recovered their ability to migrate like the WT BMDCs, while migration of the WT BMDCs was not affected (Fig. 3D).

### NO-GC1 deficiency leads to enhanced cAMP accumulation in BMDCs and promotes T-cell polarization towards Th1 in a cGMP-dependent manner

**Figure 4:**
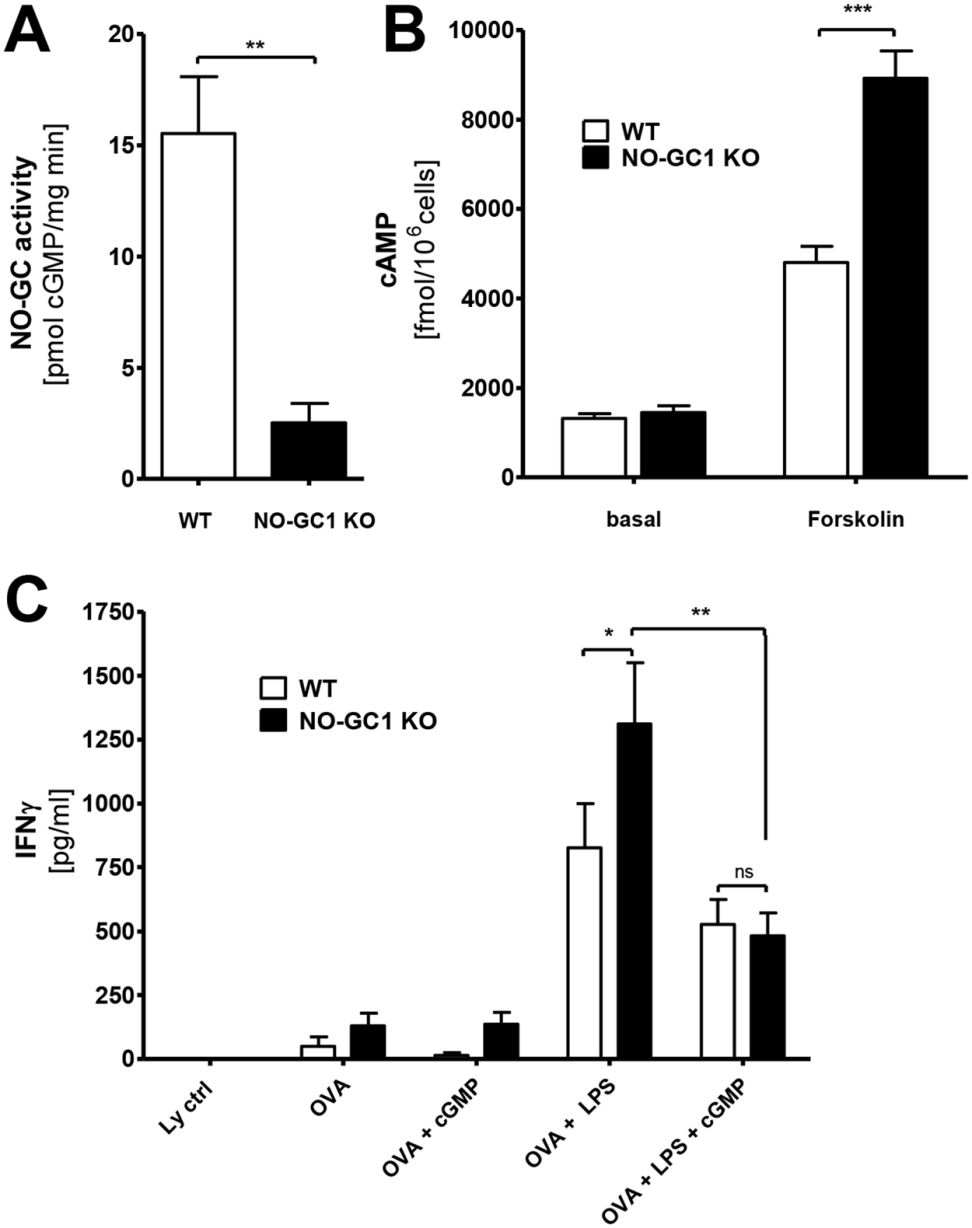
BMDCs from NO-GC1 mice display increased cAMP formation and Th1 bias. (**A**) NO-GC activity as measured in cGMP formation and (**B**) cAMP levels in lysates of WT and NO-GC1 KO BMDCs without stimulation (basal) and after treatment with the AC activator Forskolin (Fsk). (**C**) Th1 cytokine level (ELISA) in supernatant of cocultured naïve OT2 T-cells with either WT or NO-GC1 KO BMDCs with or without cGMP pretreatment. Data are mean ± SEM of n = 2 (**B**) or n ≥ 3 (**A** + **C**) independent experiments. Mann-Whitney test (**A**), One-way ANOVA with subsequent Dunn’s post test (**B**) or two-way ANOVA with subsequent Bonferroni’s post test (**C**) were performed. *P < 0.05, **P < 0.01, ***P < 0.001.

The results shown so far indicate a reduced competence of the DCs to prime T-cells towards a Th2 in favor of a Th1 phenotype due to NO-GC1 deficiency. Hence, we first studied the activity of NO-GC in BMDC homogenates. The WT BMDCs showed a distinct cGMP-forming activity, whereas the NO-GC1 KO BMDCs failed expectedly to generate cGMP (Fig. 4A). Since the polarization of T-cells was recently found to depend on cAMP signaling in the DCs^10^, we analyzed forskolin (Fsk)-triggered cAMP formation in the BMDCs. The LPS-activated BMDCs of the NO-GC1 KO mice showed a significant increase of cAMP concentration upon Fsk treatment, which was about twice as high as in the WT BMDCs, whereas the basal cAMP level (not Fsk-stimulated BMDCs) was unaltered (Fig. 4B).

We analyzed the uptake of FITC-coupled OVA because T-cell priming may depend critically on the phagocytic capacity of the (BM)DCs. We found that the amount of the phagocytosed FITC-coupled OVA per cell was not altered in the NO-GC1 KO (Supplementary Fig. 4). We next cocultured OVA-pulsed BMDCs of either WTs or NO-GC1 KOs with splenic T-cells of OVA-responsive OT II mice to analyze the T-cell priming capacity with respect to Th1/Th2 immunity. The BMDCs of the NO-GC1 KO induced a significantly stronger Th1 phenotype with a 1.5-fold increase in IFNγ secretion compared to WT, whereas Th2 cytokines (IL4, IL13) were undetectable in our setting (Fig. 4C). We treated the BMDCs with the cell-permeable cGMP analog 8-Br-cGMP prior to coculture with the OT2 T-cells to test whether this pro-Th1-inductive phenotype is a result of the lack of cGMP in the NO-GC1 KO. Intriguingly, the cGMP treatment adapts secretion of IFNγ to the WT level, whereas the IFNγ production in cocultures with WT BMDCs was not notably affected by the addition of cGMP (Fig. 4C).

### BMDCs express PDE2, which only degrades cAMP upon cGMP-dependent activation

**Figure 5:**
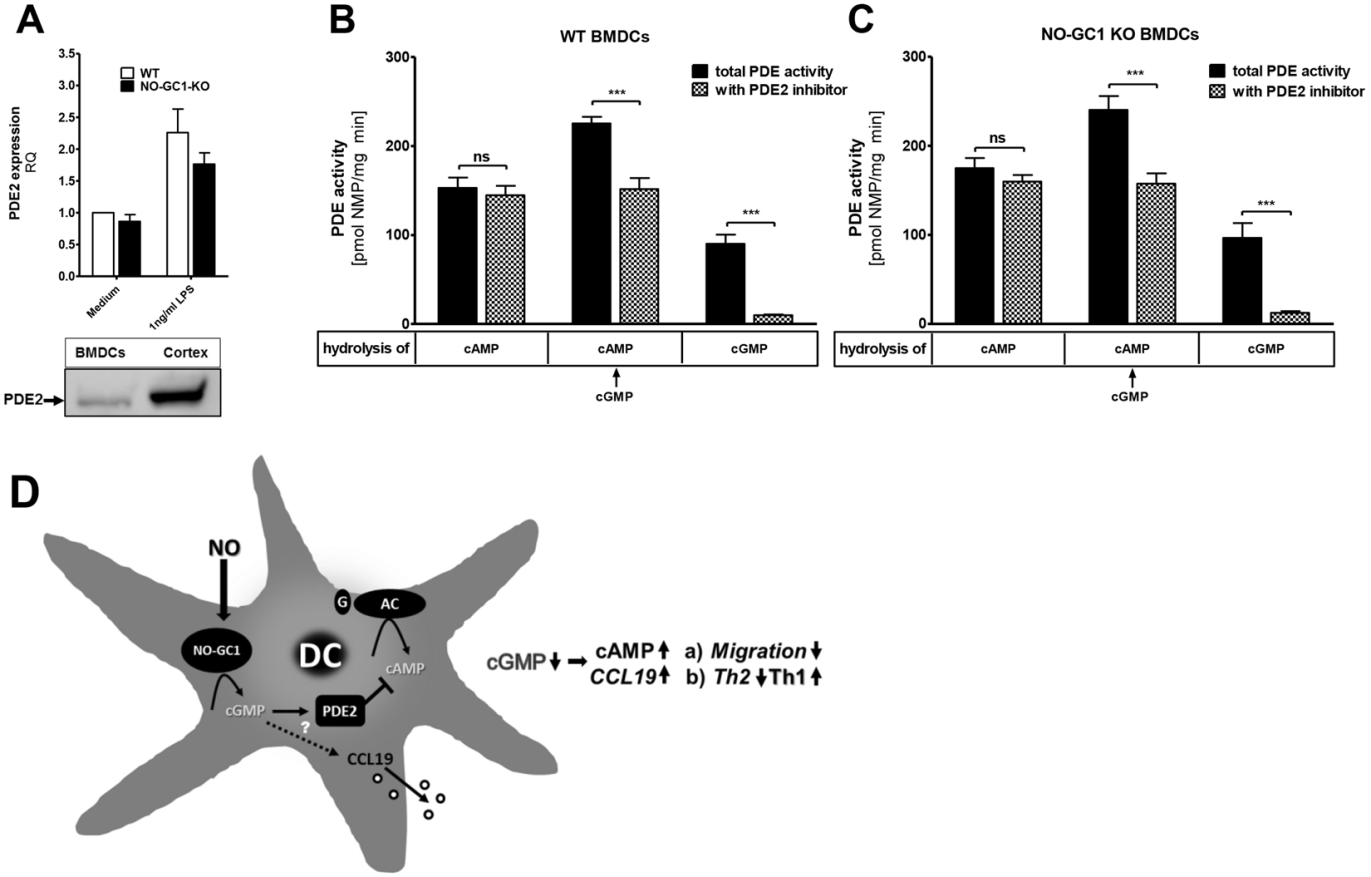
PDE2 expression and activity in BMDCs. (**A**) qPCR analysis of PDE2 expression in WT and NO-GC1 KO BMDCs and western blot of PDE2 in WT BMDCs and Cortex as a positive control (full-length blot is shown in the supplement: Supplementary Fig. 5). The relative quantification level (RQ) is based on comparison with WT “medium” (set as RQ = 1). (**B**,**C**) Activity of total PDEs and after inhibition of PDE2 in DC homogenates of WT (**B**) and NO-GC1 KO (**C**) as measured in cyclic nucleotide (NMP) turnover (=hydrolization) after supplementation of cAMP and/or cGMP. (**D**) Schematic hypothetical model of the NO-signaling regulating migration and T-cell polarization. The crosstalk of NO-GC1-derived cGMP and cAMP via PDE2 in (BM)DCs and the consequences on and T-cell fate. The second messenger cGMP is generated by the NO-activated NO-GC1. The cyclic nucleotide degrading PDE2 is activated upon cGMP-binding and hydrolyses cAMP which is generated by the Adenylat Cyclase (AC). The cAMP level within the (BM)DC biases the Th phenotype and is balanced by cGMP, with low/missing cGMP resulting in higher cAMP levels promoting Th1 differentiation and a high cGMP lowering the cAMP levels, thereby favoring Th2 differentiation. Lack of NO signaling enhances CCL19 production, which accounts for the impaired migration. Data are mean ± SEM of n = 3 (**A**) and n = 4 (**B**,**C**) independent experiments. One-way ANOVA with subsequent Dunn’s post test (**A**) or two-way ANOVA with subsequent Bonferroni’s post test (**B**,**C**) were performed. *P < 0.05, **P < 0.01, ***P < 0.001.

Our findings so far provide evidence that the reduced priming of a Th2 in favor of a Th1 phenotype is the consequence of a deregulated cAMP signaling caused by the lack of cGMP formation in the NO-GC1 KO (BM)DCs. Here, we speculated that PDE2 might bridge the cGMP and cAMP signaling, since cGMP-activated PDE2 drives the degradation of cAMP. Therefore, we analyzed the occurrence of PDE2 in the BMDCs. The expression of PDE2 mRNA upon LPS-stimulation increased about twofold independent of the genotype, and the transcript levels did not significantly differ between WT and NO-GC1 BMDCs. The expression of PDE2 protein was confirmed by Western blot and is detectable in LPS-stimulated BMDCs (Fig. 5A; full-length blot: Supplementary Fig. 5).

We next investigated the cAMP-degrading activity in the BMDC homogenates of either WT or NO-GC1 KO at a substrate concentration of 1 µM cAMP (Fig. 5B,C). Cyclic AMP degrading activity was comparable in BMDCs of WT and NO-GC1 KO mice. Inhibition of PDE2 with BAY60-7550 (20 nM) did not affect it indicating that PDE2 is not active under the conditions tested. Since PDE2 is activated by cGMP, we next measured cAMP hydrolysis in the additional presence of cGMP (1 µM). In the presence of cGMP, the cAMP-degrading activity was increased about 1.5-fold and the PDE2 inhibitor abolished this increase. Thus, PDE2-dependent cAMP hydrolysis was only detectable after cGMP activation of PDE2. In the absence of cGMP, PDE4 was identified as the major cAMP-degrading PDE being responsible for about 60% of the cAMP hydrolysis at substrate concentration of 1 µM cAMP (Supplementary Fig. 6) whereas a contribution of PDE3 was not detectable (substrate 0.1 µM cAMP). In regards of cGMP-degrading activity, PDE2 was identified as the major PDE in BMDCs as inhibition of PDE2 nearly abrogated cGMP hydrolysis (90%) in both genotypes. Thus, expression of PDE2 is similar in WT and NO-GC1 BMDCs and the enzyme hydrolyzes cAMP exclusively upon addition of cGMP *in vitro*. Here, we suggest that the accumulation of cAMP observed in the NO-GC1 KO BMDCs is a consequence of the lack of cGMP which is essential for the activation of the PDE2.

## Discussion

The maturation of dendritic cells and its regulation determines the magnitude and type of T-cell response. As allergic asthma is characterized by a hypersensitive Th2 biased reaction, the identification of signaling pathways which balance the immune response of T-cells is highly relevant for understanding the disease and the development of therapeutic intervention. Here, we demonstrate for the first time that NO-mediated signaling via NO-GC1 is involved significantly in the immunopathology of allergy, since it regulates two key features of dendritic cells, namely migration and T-cell polarization. Interestingly, the observed shift towards Th1 immunity resulted in reduced Th2 immunity with an overall reduction in eosinophilic inflammation and goblet cell metaplasia. Although IL13 as one known mediator of AHR was reduced in NO-GC1 KO mice, the response to methacholine was comparable to WT mice. One explanation for this would be that the AHR observed in NO-GC1 KO mice results from IFNγ produced in consequence of the Th1 response^34^.

In this study we have measured T-cell polarization by detecting the specific cytokines IL5 and IL13 for Th2 and IFNγ for Th1 as biomarkers. One additional marker that was measured to proof the increased polarization of Th1 cells is the transcription factor Tbet. This factor shows increased expression in T-helper cells of NO-GC1 KO mice after systemic sensitization with OVA/Alum further supporting the idea of increased Th1 polarization.

Inflammatory disorders are often accompanied and maintained by the generation of NO, which causes oxidative stress and exerts pro-inflammatory action if present in high concentrations^30,35^. On the contrary, lower concentrations were also found to act in an anti-inflammatory way, probably via NO-GC/cGMP signaling^36,37^. Additionally, there is already evidence from literature that NO and NO-mediated signaling has a strong immune regulatory role and influences Th bias^18,19,38^. Mice deficient in NO-synthesis by iNOS, especially in the context of infection, were shown to exhibit an enhanced Th1 response with higher IFNγ and less IL4 secretion^38–40^. The induction and determination of T-cell fate requires APCs, especially DCs, which themselves depend on activation of, for example, pattern recognition receptors by microbial products, such as LPS. After receiving LPS as an inducer for maturation of the DCs, the expression pattern of their chemokine receptors change. This leads especially to the upregulation of CCR7/CD197^5^, rendering the DCs to be responsive to the chemoattractants CCL19 and CCL21, both guiding the DCs to the draining lymphoid organs. The chemokine CCL19 is expressed by the DCs themselves upon LPS-mediated activation and was found to contribute essentially to further maturation of the DCs^41^. In addition to the maturation of the DCs, LPS also induces the generation of NO, which acts on the DCs in an autocrine and paracrine manner^12^.

Intriguingly, the lack of NO-GC1 in the DCs resulted either in a generally reduced T-cell response or in a shift in Th2 bias in favor of Th1, which, in turn, means that the NO signaling in DCs strengthens and modifies the adaptive immune response in allergic disease. The investigation of the migrational properties of the DCs demonstrated clearly the inability to migrate in response to CCL19 entirely, although CCR7 surface expression is not affected in NO-GC1 deficient DCs. The impaired migration observed in the NO-GC1 KO DCs arises from an enhanced endogenous production of CCL19, as its neutralization ameliorates their locomotion significantly. Since binding of CCL19 to CCR7 results in the internalization of the receptor^42,43^, the elevated CCL19 production in the NO-GC1 KO DCs might either saturate the CCR7 repertoire or enwrap the cell thereby desensitizing the DCs to respond to external chemokine, as the latter is supported by Hansen and colleagues^44^. We conclude here, that the impaired migration accounts for the dampened T-cell response observed in the mice which received the NO-GC1 KO BMDCs. The onset of expression of CCL19 is known to depend on TLR signaling by transcriptional activation via NF-κB^45,46^. Our results imply that NO signaling represses TLR-mediated expression of CCL19. One promising candidate in this context is the suppressor of cytokine signaling 1 (SOCS1), which targets the NF-κB subunit p65 for degradation^47,48^ and was recently found to be regulated by NO^49^.

Although there is a hint from the literature that NO-mediated signaling interferes with the proper differentiation of DCs *in vitro* leading to phenotypical changes^50^, the deficiency of NO-GC1 in BMDCs alters neither the amount of CD11c^+^ cells nor the expression of the surface markers MHCII, CD86 and OX40L, suggesting that the differentiation of DCs seems to be unaffected. The shift in Th bias observed in either the OVA-model or the adoptive transfer of NO-GC1 DCs is a consequence of a modified T-cell activation by DCs due to NO-GC1 deficiency.

It was recently demonstrated that G protein-dependent cAMP formation in DCs affects Th2 bias. The authors showed that the CD11c-specific deletion of Gαs and the subsequent decrease in cAMP results in spontaneous Th2 immunity corresponding to an allergic phenotype^10^. We interpret the enhanced cAMP accumulation induced by the AC activator Fsk in our KO BMDCs together with the attenuated Th2 response in the NO-GC1 KO mice as in line with the study of Lee *et al*.^10^. Yet, because of the very low cAMP-forming capacity of our BMDCs compared to FACS-sorted DCs used in the study of Lee *et al*.^10^, we failed to increase cAMP upon activation of G-protein-coupled receptors. In another set of experiments in which BMDCs were co-cultured with OT2 T-cells, we found an increased Th1 response in NO-GC1-deficient BMDCs. Noticeable, cGMP addition decreased the Th1 response to WT-like levels. Together, these data are compatible with the assumption that cGMP and cAMP have an impact in the regulation of the Th bias by the BMDCs. Unfortunately, the Th2 cytokines IL4 and IL13 were not detectable in our coculture system, which might be due to the already low Th2 responsiveness of C57bl6 mice, especially the NO-GC1 KO ones.

A promising candidate to bridge between the cAMP and cGMP pathway is PDE2, which hydrolyzes cAMP preferentially upon activation via cGMP^30,35^. Indeed, we detected PDE2 expression in BMDCs, which was comparable in WT and NO-GC1 KO. Moreover in *in vitro* experiments, we clearly demonstrated that activation of PDE2 by cGMP is required to induce measurable cAMP hydrolysis. Thus, the loss of the cGMP-forming activity in KO BMDCs most likely attenuates the ability of PDE2 to regulate cAMP signaling.

Nitric oxide is well characterized as a key effector molecule in the defense of intracellular pathogens, such as bacteria, viruses or parasites^29,51,52^, but little is known about the mechanism of how NO affects adaptive immunity. Here, our data advance the knowledge about the role of NO and downstream signaling on DC function thereby modulating the magnitude and balance of Th1/Th2 responses. In the context of allergic disease, NO-dependent cGMP signaling via NO-GC1 is triggered by the continuously produced NO^53^. This NO-signaling might support the rapid trafficking of DCs to lymphoid organs as well as maintaining or even promoting Th2 response to counteract the Th1-mediated microbicidal action, probably by PDE2-driven limitation of cAMP signaling. Therefore, selective spatiotemporal modulation of the NO/cGMP cascade, in terms of DC trafficking and restoring the “optimal” balance of Th1 and Th2 response, may be a meaningful trial to prevent exacerbation of allergic disease.

## Materials and Methods

### Animals

For the adoptive transfer experiments female six-week-old C57/Bl6Rj (Janvier) were purchased and then adapted to the animal facility for 14 days prior to the experiments. Furthermore, female NO-GC1 KO mice (7–8 weeks old) lacking the α_1_ subunit of the heterodimeric NO-GC1 (α_1_β_1_) backcrossed to C57Bl/6Rj background for more than 12 times (>N12 generation) and their wildtype littermates were used for the induction of allergic airway inflammation and the generation of BMDCs^54^. The TCR-transgenic OVA-responsive OT II mice which we used for the coculture experiments (at 10–12 weeks of age) were bred in our facility. The animals had access to food and water ad libitum. All experimental procedures in this manuscript were approved by the animal ethics committee at the “Landesamt für Natur, Umwelt und Verbraucherschutz” (LANUV) Nordrhein-Westfalen, Germany (approval number: Az. 84-02.04.2013.A112) and were performed in accordance with all relevant guidelines and regulations.

### Sensitization and induction of allergic airway response with OVA

Wildtype and NO-GC1 KO Mice (7–8 weeks old) were sensitized and challenged with chicken OVA grade V (Sigma, St. Louis, MO). Briefly, the mice were sensitized by an intraperitoneal injection (100 μl) of 20 μg OVA emulsified in 2 mg Imject Alum (Al [OH]3/Mg [OH]2; Pierce, Rockford, IL) on days 0, 14 and 21. Subsequently, mice were challenged with an OVA aerosol every week on two consecutive days over a period of eight weeks. The OVA aerosol was generated using a PARI-Boy aerosol generator (PARI, Starnberg, Germany) from a 1% (wt/vol) OVA solution in saline for 30 min.

### Airway responsiveness (AHR)

24 h after the last aerosol challenge, airway hyperresponsiveness (AHR) to methacholine aerosol (0, 6, 12, 25 and 50 mg/ml, Sigma) was evaluated in conscious, unrestrained mice using whole-body plethysmography (Buxco Electronics) by measuring the enhanced pause (Penh). Penh values were expressed as percent increase above baseline.

### Generation of bone marrow-derived cells *in vitro*

Bone marrow-derived cells (BMDCs) were generated by cultivating BM cells in the presence of granulocyte-macrophage colony-stimulating factor, as described elsewhere^31,55^. On day eight, the cells were pulsed with 100 µg/ml OVA free of LPS (Hyglos GmbH, Germany). Chromatographically purified E. coli-lipopolysaccharide was used at a concentration of 1 ng/ml for activation of the BMDCs at day nine^6,56^. Approximately 90% of these cells (from WT and NO-GC1 KO) express CD11c. Furthermore the cells co-express CD11b and MHC class II molecules (Supplementary Fig. 2). Therefore the phenotype of the *in vitro* generated cells resembles inflammatory DCs of the lung.

### Flow cytometric analysis

Analysis of DC-specific surface antigens was performed by flow cytometry (Partec, Münster, Germany). The following fluorescein-isothiocyanate- (FITC) or phycoerythrin- (PE) conjugated rat anti-mouse monoclonal antibodies (mAbs) were used for direct labeling of the cells: anti-CD11c-PE (clone N418) from BDbioscience, anti-CD86-FITC (clone GL1), anti-MHCII-FITC (clone M5/114.15.2) and anti-OX40L-PE (clone RM134L) eBioscience; anti-CD197(CCR7)-PE (Milteny Biotec, Bergisch Gladbach, Germany). Intracellular staining of transcription factors was also analyzed by flow cytometry. Therefore spleens of mice were removed after systemic sensitization with OVA/Alum. Red blood cells were lysed by hypotonic shock and afterwards T-helper cells were isolated with a MACS negative isolation kit (Miltenyi, Bergisch Gladbach, Germany). Cells were then stained for CD4 and subsequently fixed and permeabilized with a commercial buffer kit (Thermo Fisher Scientific). The transcription factor T-bet was stained with a PerCP-Cy5.5 labeled monoclonal antibody (clone eBio4B10, Thermo Fisher Scientific). Cells were analyzed on a FACS Canto II (BDbiosciences, Heidelberg, Germany).

### Sensitization of mice with OVA-pulsed BMDCs and airway challenge

The WT mice were anesthetized with a mixture of Ketamin 65 mg/kg bodyweight and Rompun 13 mg/kg bodyweight for sensitization with BMDCs of either WT or NO-GC1 KOs via the airways. An amount of 10^6^ OVA-pulsed BMDCs were administered intranasally in 50 µl PBS. Mice were challenged for the induction of airway inflammation on days five, six and seven with 1% OVA aerosol for 30 min using a PARI-Boy aerosol generator (PARI, Starnberg, Germany). Controls did not receive BMDCs, but were treated with OVA aerosol. Mice were sacrificed three days after the third OVA challenge for analysis (Fig. 2A).

### Bronchoalveolar lavage (BAL)

Three days after the last OVA challenge, lungs were lavaged via a tracheal cannula with 2 × 1 ml PBS, and the leukocytes in the lavage fluid were counted. After centrifugation, BAL fluid was frozen for further analysis. Cytospin slides of BAL cells were stained with a fast staining procedure (HAEME-Schnellfärbung, Labor + Technik Eberhard Lehmann, Berlin, Germany), according to the manufacturer’s instructions. The percentages of eosinophils, lymphocytes and macrophages in the BAL samples were determined by light microscopy. An example showing the cells that were counted is shown in Supplementary Fig. 7. At least 300 cells per sample were differentially counted by a blinded investigator.

### Histology

Lungs were fixed by inflation with 1 ml of 4% buffered PFA via a tracheal tube. Further fixation was carried out by incubation of the lungs in the same fixation medium for 24 h. Afterwards lungs were embedded in paraffin and sliced into 5 µm sections with a microtome. After deparaffinization, slices were stained for goblet cells with the periodic acid-Schiff (PAS) method or with hematoxylin and eosin to evaluate the total lung inflammation. Stained slices were analyzed using a light microscope (Olympus BX40, Olympus; Hamburg, Germany). Digital image analysis was performed using ImageJ (National Institutes of Health). Mucus-producing cells in PAS-stained tissue were counted, the length of the airway epithelium was measured and the number of positive cells per µm was calculated.

### *In vitro* cytokine production of mouse lymphocytes

Spleens and lungs were harvested and single cell suspensions were prepared from spleens by mechanical disruption or from lungs by digestion with collagenase type III from Clostridium histolyticum (0.5 mg/ml, Sigma, St. Louis, MO). Erythrocytes were lysed by hypotonic shock. Lymphocytes were then cultured at a concentration of 10^7^/ml in complete tissue culture medium (RPMI 1640 with 10% fetal calf serum, 2 mM L-glutamine, 100 U/ml penicillin, 100 µg/ml streptomycin, all from Biochrom, Berlin, Germany). Cultures were treated with 100 µg/ml OVA (Hyglos GmbH, Germany) or PBS (control) for restimulation. After 48 h of culture, the supernatants were collected and stored at −80 °C until analysis.

### Measurement of cytokines in cell culture supernatants and BAL fluid

Levels of IL13, IL5 and IFNγ were assessed by using optEIA kits (BD Biosciences, Heidelberg, Germany). The TGF-ß1, IL4, IL10 and IL17A was measured with a commercial ELISA set (eBioscience, San Diego, USA), according to the manufacturer’s instructions.

### Measurement of OVA-specific IgE

Levels of OVA-specific IgE in serum or BAL fluid were determined by ELISA. Briefly, sample wells of a Nunc maxisorb ELISA plate (Nunc, Wiesbaden, Germany) were coated with 5 μg/ml OVA overnight and then blocked with 1% bovine serum albumin. After incubation with diluted samples, bound OVA-specific antibodies were detected with biotinylated rat anti-mouse IgE (clone R35–72, BD Biosciences). The biotinylated antibody was detected by horseradish peroxidase conjugated extravidin (Sigma, St. Louis, MO) and a TMB substrate kit (eBioscience). A standard curve was generated with a commercial anti-OVA IgE standard (clone 2C6; AbD Serotec, Puchheim, Germany).

### Measurement of OVA-specific IgG1

Levels of OVA-specific IgG1 in serum or BAL fluid were determined by ELISA. Briefly, sample wells of a Nunc maxisorb ELISA plate (Nunc, Wiesbaden, Germany) were coated with 5 mg/ml OVA at 4 °C overnight and then blocked with 5% MMP in Tris buffer. After incubation with diluted samples, bound OVA-specific antibodies were detected with rat anti-mouse IgG1 (clone X56, BD Biosciences). The alkaline phosphatase conjugated antibody was detected with pNPP substrate (4-Nitrophenyl phosphate 1 mg/ml in diethanolamine). A standard curve was generated with a commercial anti-OVA IgG1 standard (clone OVA-14 ascites, Sigma, St. Louis, MO).

### *In vitro* Migration assay

BMDCs were generated as described above. On day 8 BMDCs were activated with 1 ng/ml LPS for 24 hours. On day 9 BMDCs (1 × 10^5^ cells in 200 µl Medium) were loaded in the upper chamber of a Transwell insert (5 μm pore size; Sarstedt, Nümbrecht, Germany), while Medium (600 μl) with or without CCL19 (12,5 ng/ml, Immunotools, Friesoythe, Germany) was added to the lower chamber. After 2 h at 37 °C, migrated cells were harvested from the lower chamber and counted in a Neubauer chamber. For neutralizing endogenous CCL19, BMDCs were treated with anti-CCL19/MIP-3 beta polyclonal antibody (15 µg/ml; R + D, Minneapolis, US) on day 8 for 24 hours. The addition of LPS and anti-CCL19 was carried out simultaneously. On day 9 BMDCs were detached and washed twice with fresh medium to remove the anti-CCL19 antibody prior to the transwell-assay.

### Antigen Uptake assay

BMDCs were generated as described above. On day 6 BMDCs (2 × 10^5^ cells in 200 µl Medium) were incubated with FITC labeled-chicken OVA (FITC-OVA; both Sigma, St. Louis, MO) as a model antigen in a final concentration of 0; 1,5; 7,5; 15 and 75 µg/ml for 1 h at 37 °C. Afterwards cells were washed 3 times, fixed and the fluorescence of internalized FITC-OVA was measured in the flow cytometer (Partec, Münster, Germany). Fluorescence values were reported as mean fluorescence intensity (MFI).

### Western blot

The LPS-activated BMDCs were detached, centrifuged (800 × g, 5 min, 4 °C) and cell pellets were frozen in liquid nitrogen. In order to obtain homogenates of BMDCs, frozen cell pellets (~10^7^ cells) were homogenized in 300 µl of buffer (50 mmol/L TEA/HCl, 50 mmol/L NaCl, 2 mmol/L DTT, 0.2 mmol/L benzamidine, 0.5 mmol/L phenylmethylsulfonyl fluoride, 1 µmol/L pepstatin A; pH 7.4, 4 °C) using a glass/glass homogenizer (900 rpm). Protein concentrations were determined using a Bradford assay (Bio-Rad, Germany). Western blotting was performed, as described previously^54^. Anti-PDE2A (sc-17228, Santa Cruz, Heidelberg, Germany) was used in a 1:1,000 dilution.

### Determination of NO-stimulated GC activity

The NO-stimulated GC activity was determined in homogenates of BMDCs (3 µg) by repetitive addition of 100 µmol/L DEA-NO (at time point 0, 6, 12, 18 and 24 min) in the presence of GTP (0.25 mmol/L) and Bay 41–2272 (10 µmol/L) for 30 min at 37 °C. The cGMP forming was quantified by RIA, as described previously^54,57^.

### Measurement of PDE activity

The PDE activity was measured by the conversion of [^32^P]cAMP or [^32^P]cGMP (synthesized from [α-^32^P]ATP or [α-^32^P]GTP using purified NO-GC) to either adenosine or guanosine and [^32^P]phosphate in the presence of alkaline phosphatase (Sigma, St. Louis, MO) at 37 °C for 5 min. Reactions mixtures (0.1 ml) contained BMDC homogenates (5 µl, ~5–10 µg protein) [^32^P]cAMP or [^32^P]cGMP (~2 kBq), 1 µmol/L cAMP or cGMP, 12 mmol/L MgCl_2_, 3 mmol/L DTT, 0.5 mg/ml BSA, 2 U of alkaline phosphatase and 50 mmol/L TEA/HCl, pH 7.4. Reactions were stopped by the addition of 900 µl ice-cold charcoal suspension (30% activated charcoal in 50 mmol/L KH_2_PO_4_, pH 2.3). After pelleting the charcoal by centrifugation (12,000 × g, 4 min), [^32^P]phosphate was measured in the supernatant. Bay 60–7555 at 20 nmol/L was added to the measurements to determine the PDE activity ascribed to PDE2. Because PDE2 is activated by cGMP, cAMP hydrolysis (1 µmol/L substrate) was also measured in the presence of 1 µmol/L cGMP. PDE4-dependent cAMP hydrolysis (1 µmol/L substrate) was determined using the inhibitor Rolipram (10 µmol/L). PDE3-dependent cAMP hydrolysis (0.1 µmol/L substrate) was determined using the inhibitor Cilostamid (1 µmol/L). The PDE assays were carried out in triplicates.

### Determination of cAMP content in BMDCs

The LPS-activated BMDCs (24-well plates at 10^6^ cells per well) allowed equilibrating for 30 min in HEPES buffer (154 mM NaCl, 5.6 mM KCl, 2 mM CaCl2, 1 mM MgCl2, 3.6 mM NaHCO3, 10 mM HEPES, pH 7.4, 5.6 mM glucose, 37 °C) and were stimulated by Fsk (10 µmol/L, 15 min, 37 °C). Cells were lysed by exchanging the HEPES buffer with ice-cold 70% (v/v) ethanol. After centrifugation (20,000 × g, 15 min, 4 °C), the supernatants were dried at 95 °C and the cGMP contents were measured by RIA^54,57^.

### OVA-specific immune responses upon a coculture system

The BMDCs from NO-GC1 KO and WT mice were generated as described above with a few modifications. On day seven, the cells were pulsed with 50 µg/ml OVA free of LPS (Hyglos GmbH, Germany). The BMDCs were activated on day eight with chromatographically purified E. coli-lipopolysaccharide at a concentration of 1 ng/ml for 24 h and then cocultured (1 × 10^5^ cells) with OT 2 T-cells from the spleen of OT 2 mice in complete RPMI medium (ratio 1:20) for 48 h^56^. The OT 2 mice express the mouse alpha-chain and beta-chain T cell receptor that pairs with the CD4 coreceptor and is specific for ovalbumin.

The cGMP-stimulation of the BMDCs was performed with 8-Bromoguanosine 3′,5′-cyclic monophosphate (8-Br-cGMP, 1 µM, Sigma, St. Louis, MO) on day eight with LPS simultaneously.

The LPS-activated cells (24 h) were treated with Fsk (10 µM) for 15 min prior to coculture with OT2 T-cells for the activation of AC in the BMDCs. The BMDCs were washed twice with PBS to remove Fsk prior to coculture.

Supernatant was collected for cytokine measurement with ELISA.

### Real time polymerase chain reaction

Total RNA was extracted from cultured cells with TRIzol® reagent (Invitrogen). A reverse transcription kit (Applied Biosystems) was used to construct the template cDNA for real-time polymerase chain reaction (RT-PCR). Quantitative PCR was performed using the SYBR Green RT-PCR method. Quantitative RT-PCR was performed on an ABI *Step One* PCR instrument (Applied Biosystems, Foster City, CA) using three-stage program parameters provided by the manufacturer as follows: 2 min at 50 °C, 10 min at 95 °C and then 40 cycles of 15 s at 95 °C and 1 min at 60 °C. Specificity of the amplification product produced was confirmed by examination of dissociation reaction plots. A distinct single peak indicated that a single DNA sequence was amplified during PCR. Samples obtained from three independent experiments were used for analysis of relative gene expression data using the RQ (2^−ΔΔ*C*T^). The housekeeping gene hypoxanthine-guanine phosphoribosyltransferase (HPRT) served as a reference gene/endogenous control.

Primer used for real time polymerase chain reaction:

CCL19_forward 5′-ATG TGA ATC ACT CTG GCC CAG GAA-3′

CCL19_reverse 5′-AAG CGG CTT TAT TGG AAG CTC TGC-3′

PDE2_upper 5′-AAG GGC TGG AAG ACC ACC A-3′

PDE2_lower 5′-CAT CTC CAT CGG TCG GTT G-3′

HPRT_forward 5′-GAT TCA ACT TGC GCT CAT CTT A-3′

HPRT_reverse 5′-GTT GGA TAC AGG CCA GAC TTT GTT G-3′.

### Statistics

All data were analyzed by a one-way ANOVA Kruskal–Wallis test and a subsequent Dunn’s test, two-way ANOVA with subsequent Bonferroni’s post test or Mann-Whitney test as indicated. The groups treated were compared with the untreated group and the WTs compared to NO-GC1 KOs using Graph Pad Prism software V.5 (La Jolla, California, USA) for analysis. Values of p < 0.05 were considered statistically significant (*p < 0.05, **p < 0.01, ***p < 0.001). Results are presented as mean ± SEM, as indicated.

## Electronic supplementary material

Supplementary Figures
