# Supplementary material for: Nitric oxide dependent signaling via cyclic GMP in dendritic cells regulates migration and T-cell polarization
Source: Sci Rep. 2018 Jul 20;8:10969. doi: 10.1038/s41598-018-29287-9 (PMC6054623; doi:10.1038/s41598-018-29287-9)
Supplement: Supplementary file 1 — Supplementary Figures [file 41598_2018_29287_MOESM1_ESM.pdf]

# Supplementary information

**Nitric oxide dependent signaling via cyclic GMP in dendritic cells regulates migration and T-cell polarization**

Stefanie Gnipp[1], Evanthia Mergia[2], Michelle Puschkarow[1], Albrecht Bufe[1], Doris Koesling[2], Marcus Peters[1]

[1] Department of Experimental Pneumology, Medical Faculty, Ruhr University Bochum, 44780 Bochum, Germany

[2] Institute of Pharmacology and Toxicology, Medical Faculty, Ruhr University Bochum, 44780 Bochum, Germany

## Supplementary Figure 1

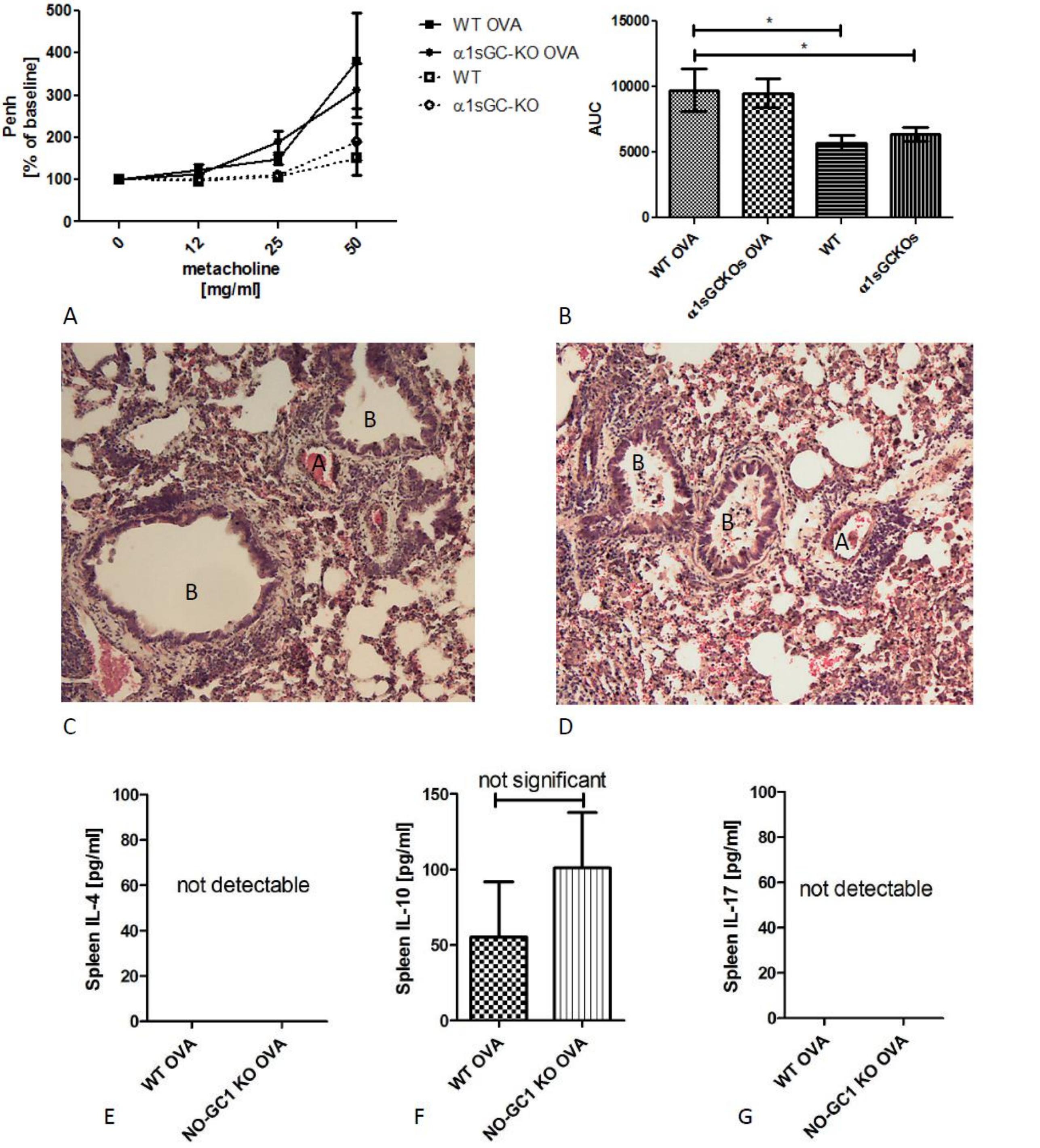


**Analysis of the allergic airway inflammation in the OVA Alum Modell.** Airway hyperreactivity (AHR) to increasing doses of methacholine was measured by whole body plethysmography. AHR was expressed as increase of Penh over baseline values (A). To

determine statistical differences between the groups of mice area under the curve (AUC) was determined for the individual Penh curves (B). AHR is significantly increased in mice sensitized and challenged with ovalbumine. Furthermore lung inflammation was determined by H&E staining of lung tissue. Representative photographs are shown from k.o. mice (C) and wild type mice (D). Bronchi are labelled with “B” and arterioles with “A”. Additional cytokine measurements in supernatants of splenocytes were done for IL-4 (E), IL-10 (F) and IL-17A (G).

## Supplementary Figure 2

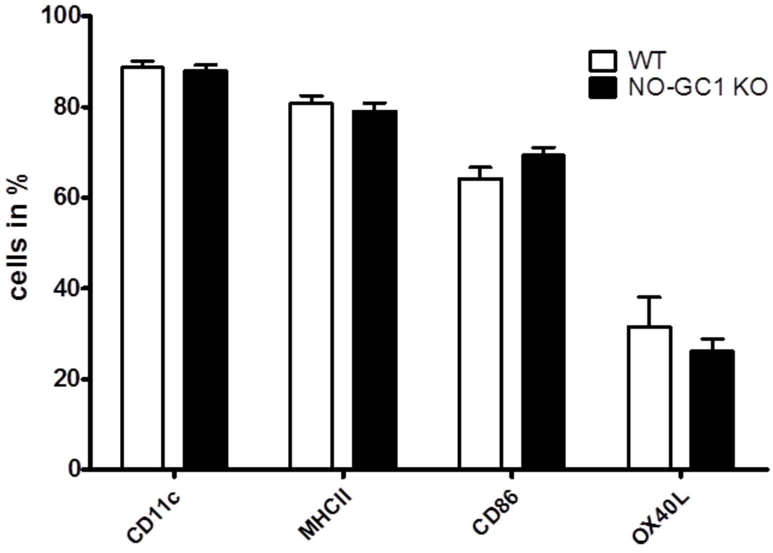


**Expression of surface markers on BMDCs.** The proportion of cells expressing CD11c, MHCII, CD86 and the OX40L was determined in FACS analysis. Data are mean ± SEM of n = 15 /group. Mann-Whitney test was performed for each marker to compare WT and KO.

**Supplementary Figure 3**

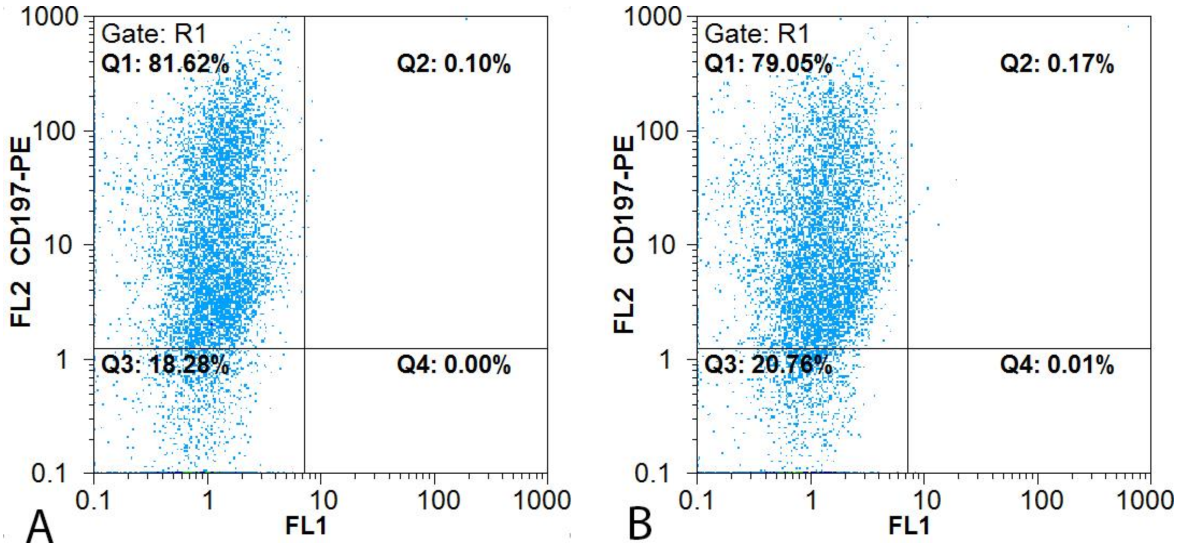


**Measurement of CD197 expression on BMDCs.** The expression of the chemokine receptor CD197 was detected on the surface of BMDC from either WT (A) and NO-GC1 KO (B) mice. The diagrams show representative dot plots from the flow cytometric analysis.

## Supplementary Figure 4

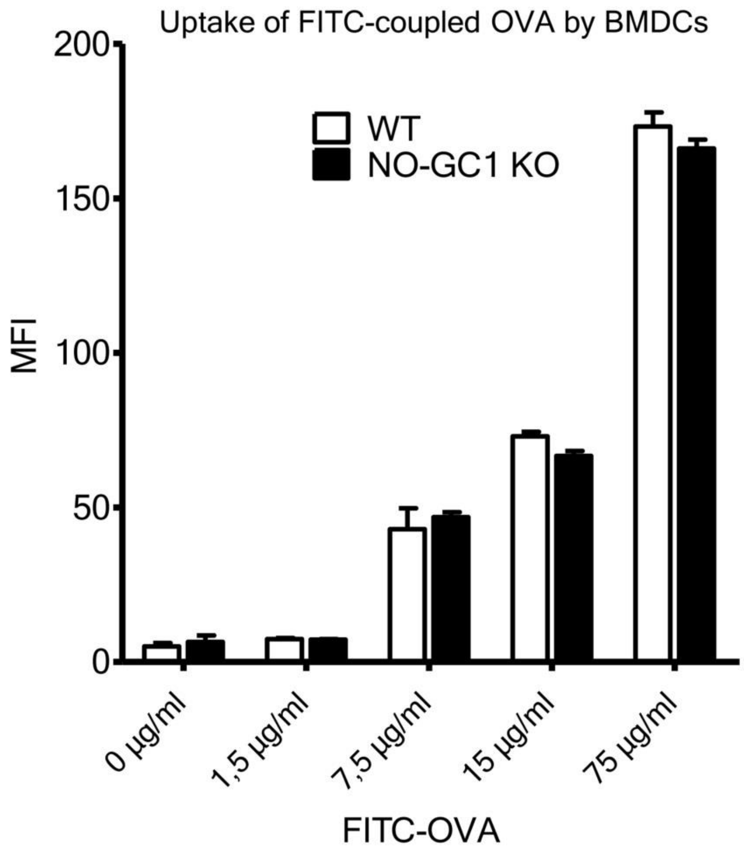


**Uptake of FITC-coupled OVA by BMDCs.** The capacity of phagocytosis is reflected by the MFI (mean fluorescence intensity) of WT and NO-GC1 KO BMDCs and was determined in FACS analysis. FITC: Fluorescein isothiocyanate. Data are mean ± SEM of n = 3 /group.

**Supplementary Figure 5**

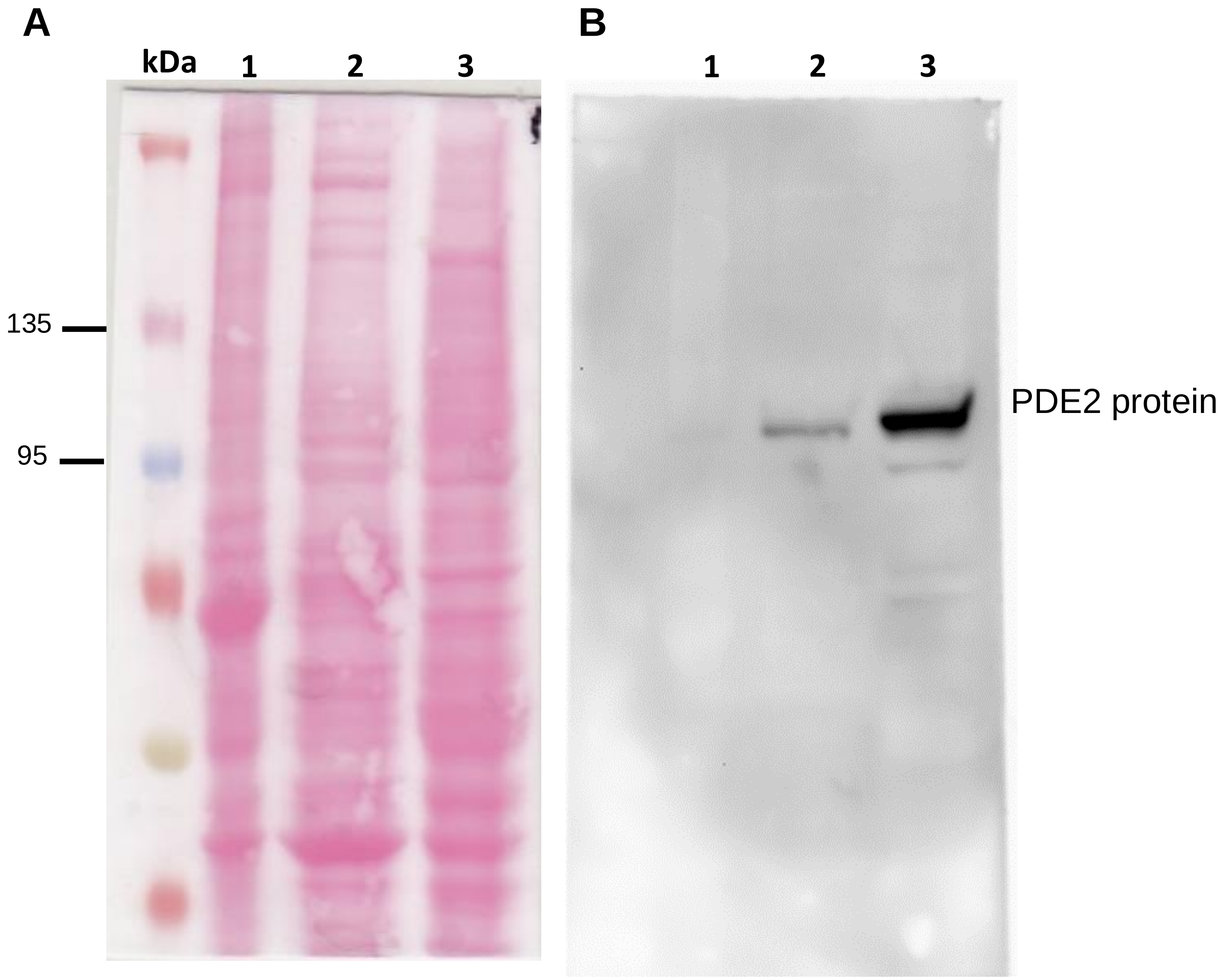


**Full-length blot of PDE2A.** (A) Ponceau S staining of the membrane. (B) Detection of the PDE2A protein (105 kDa) with a polyclonal antibody (Santa Cruz Biotechnology, sc-17228). Lane 1: cells without PDE2 as negative control (vascular smooth muscle cells); lane 2: LPS-stimulated BMDCs; lane 3: murine brain Cortex as a positive control.

## Supplementary Figure 6

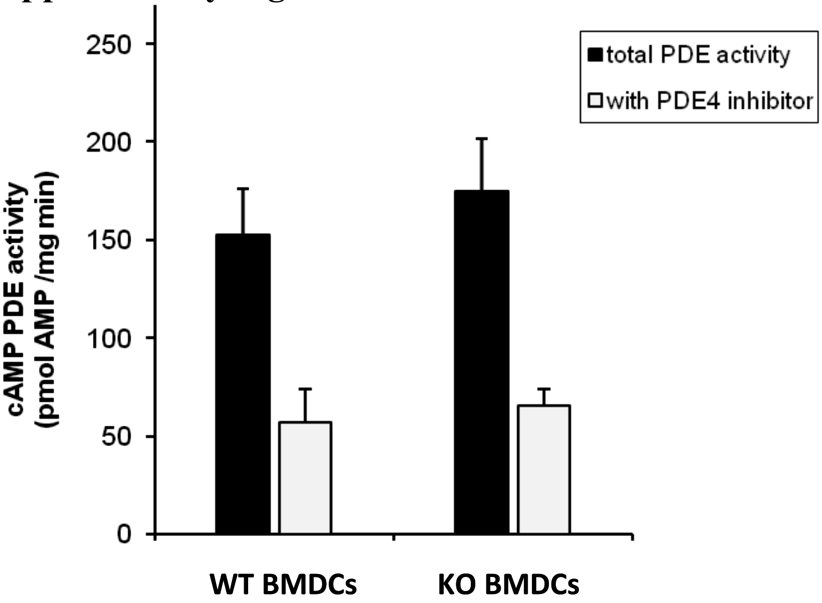


**Contribution of PDE4 to total cAMP-hydrolysing activity**. Cyclic AMP-hydrolysing activity measured in BMDC homogenates of WT and NO-GC1 KO mice at a substrate concentration of 1 µM cAMP without and with a PDE4-specific inhibitor (rolipram 10 µM).

## Supplementary Figure 7

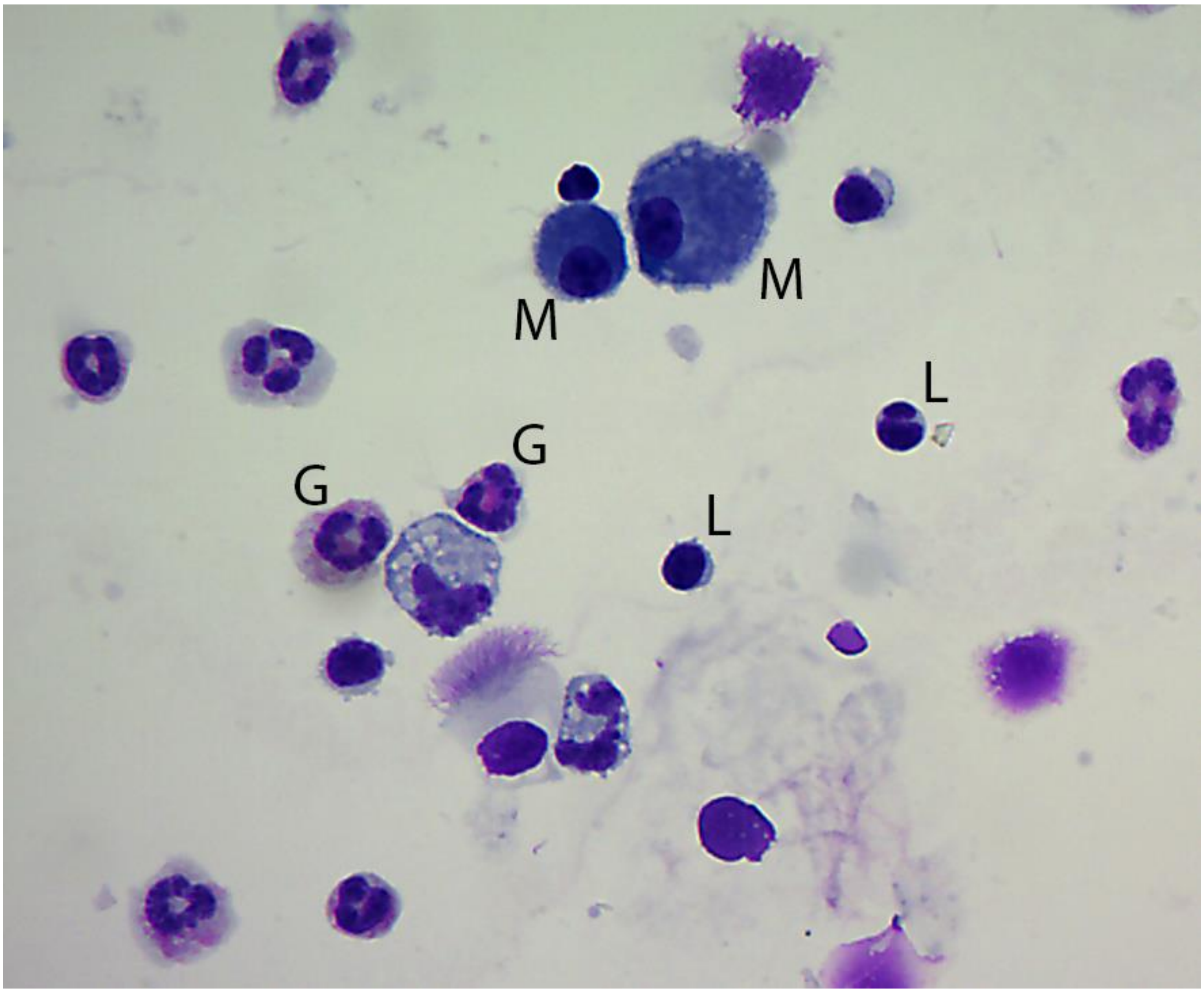


**Differentiation of BAL cells.** Three days after the last OVA challenge, lungs of mice were lavaged via a tracheal cannula. Cytospin slides of BAL cells were stained with a fast staining procedure (HAEME-Schnellfärbung, Labor+Technik Eberhard Lehmann, Berlin, Germany), according to the manufacturer's instructions. The percentages of eosinophils (G), lymphocytes (L) and macrophages (M) in the BAL samples were determined by light microscopy.
